# How tree species have modified the potentially toxic elements distributed in the developed soil–plant system in a post-fire site in highly industrialized region

**DOI:** 10.1007/s10661-024-12933-3

**Published:** 2024-08-03

**Authors:** Bartłomiej Woś, Justyna Likus-Cieślik, Marek Pająk, Marcin Pietrzykowski

**Affiliations:** Faculty of Forestry, Department of Ecological Engineering and Forest Hydrology, University of Agriculture in Krakow, al. Mickiewicza 21, 31-120 Krakow, Poland

**Keywords:** Heavy metals, Trace elements, Soil recovery, Disturbance, Bioaccumulation, Translocation

## Abstract

The biogeochemical cycles of trace elements are changed by fire as a result of the mineralization of organic matter. Monitoring the accumulation of trace elements in both the environment and the tree biomass during the post-fire (PF) forest ecosystem regeneration process is important for tree species selection for reforestation in ecosystems under anthropogenic pressure. We analyzed the soil concentrations of different groups of potentially toxic elements (PTEs), including beneficial (Al), toxic (Cd, Cr, Pb), and microelements (Cu, Mn, Ni, Zn), and their bioaccumulation in the tree species (*Pinus sylvestris*, *Betula pendula*, *Alnus glutinosa*) biomass introduced after a fire in a forest weakened by long-term emissions of industrial pollutants. The results indicated no direct threat from the PTEs tested at the PF site. The tree species introduced 30 years ago may have modified the biogeochemical cycles of the PTEs through different strategies of bioaccumulation in the belowground and aboveground biomass. Alder had relatively high Al concentrations in the roots and a low translocation factor (TF). Pine and birch had lower Al concentrations in the roots and higher TFs. Foliage concentrations and the TF of Cd increased from alder to pine to birch. However, the highest concentration and bioaccumulation factor of Cd was found in the alder roots. The concentrations of Cr in the foliage and the Cr TFs in the studied species increased from pine to birch to alder. Higher concentrations of Cu and Ni were found in the foliage of birch and alder than of pine. Among the species, birch also had the highest Pb and Zn concentrations in the roots and foliage. We found that different tree species had different patterns of PTE phytostabilization and ways they incorporated these elements into the biological cycle, and these patterns were not dependent on fire disturbance. This suggests that similar patterns might also occur in more polluted soils. Therefore, species-dependent bioaccumulation patterns could also be used to design phytostabilization and remediation treatments for polluted sites under industrial pressure.

**Supplementary Information:**

The online version contains supplementary material available at 10.1007/s10661-024-12933-3.

## Introduction

Wildfire frequency is expected to increase globally in the coming years (Jain et al., 2021), with climate change considered to be the leading cause (Abram et al., 2021; Pausas & Keeley, 2021). Other factors may include social changes related to abandoning traditional agricultural land use and afforestation (Pausas & Keeley, 2021). Fires can strongly modify ecosystems, especially the physical, biological, and chemical soil properties, including the biogeochemical cycling of elements (Certini, 2005; González-Pérez et al., 2004). Forest ecosystems are degraded after high-severity fires, and the presence and increased mobility of potentially toxic elements (PTEs) may additionally affect the vitality of trees, especially in sites under pressure from industry (Fernandez-Marcos, 2022). However, the impacts of fire on the deposition, mobilization, and fate of trace elements and other PTEs have been underinvestigated (Campos et al., 2016; Fernandez-Marcos, 2022). Potentially toxic elements may include metals toxic to animals and plants, such as trace elements (Pb, Cr, Cd), or microelements (Mn, Cu, Zn, Ni) that are important for the growth and functioning of organisms (Nagajyoti et al., 2010; Nawrot et al., 2021). Another group includes so-called beneficial elements, such as Al, which are not essential for proper functioning, but can stimulate the growth of certain plant species or are only necessary for selected species (Broadley et al., 2012). Both microelements and beneficial elements can become toxic to plants and animals when they exceed threshold concentrations (Kabata-Pendias, 2001). Potentially toxic elements typically occur in relatively low concentrations in unpolluted soils (typically below 100 mg kg^−1^ as trace elements), depending on the parent rock and mineral weathering processes (Terzano et al., 2021). However, certain PTEs may exist as trace elements in plant organs, but not in the soil (e.g., Al occurs in rocks, but it is also found as a trace element in plants, albeit its mobility is low) (Kabata-Pendias, 2001).

The burning of vegetation and soil organic horizons and the mineralization of dead organic matter as a result of large-scale fires can release a considerable amount of PTEs, which enrich the topsoil horizons (Campos et al., 2016). Through volatilization, part of the PTE pool is also released into the atmosphere as smoke particles (Ignatavičius et al., 2006). Fires also alter the mobility of the trace elements in soil (Abraham et al., 2017). One of the soil modifications caused by fire is an increase in pH due to ash accumulation (Agbeshie et al., 2022; Neary et al., 2005). Most trace elements are more available to plants at low soil pH (Zeng et al., 2011). However, elements occurring as anions in aqueous solutions (e.g., Cr) may be more soluble in an alkaline environment (Jiang et al., 2008; Reijonen & Hartikainen, 2016). During ecosystem recovery following a fire, PTEs can be incorporated into the biological cycle through their uptake by plants and microorganisms, or they can be lost through leaching into deeper soil horizons or through surface runoff (Pereira & Úbeda, 2010).

In terms of the selection of tree species for the reforestation of post-fire (PF) sites, it is important to select appropriate pioneering species because; under the conditions of degraded habitat, there are ecological factors characteristic of bare surfaces to be taken into account, such as full sunlight and high-temperature amplitudes (Castro, 2021; Dzwonko et al., 2015). In Central Europe, silver birch (*Betula pendula* Roth) and Scots pine (*Pinus sylvestris* L.) are two main pioneer species that are introduced to sites after fire disturbance (Dzwonko et al., 2015; Woś et al., 2022). After large-scale disturbances, one of the most deficient elements is N, and N-fixers, such as black alder (*Alnus glutinosa* (L.) Gaertn.), are also often introduced, although to a lesser extent (Woś et al., 2022). The bioaccumulation of PTEs, especially under the conditions of ecosystems under industrial pressure, is the second aspect to be considered in selecting the species composition for reforestation (Alahabadi et al., 2017; Bartkowiak et al., 2020). Different tree species may also affect the bioavailability of PTEs through the differing quantities and properties of their root exudates, or substances, such as organic acids, leached from the litterfall of the aboveground biomass (Evangelou et al., 2006; Mertens et al., 2007; Montiel-Rozas et al., 2016). Selection of the tree species is important for the rate of soil organic matter (quantity and quality) recovery after disturbance (Woś et al., 2023) and therefore also for the binding of PTEs in organic–mineral complexes (Bartkowiak et al., 2020). Through bioaccumulation and the subsequent translocation of various PTEs from the belowground (roots) to the aboveground (leaves) biomass components, different tree species can influence either the intensification of the biological cycling of PTEs or the exclusion of a significant pool of them from the biological cycle (Budzyńska et al., 2021; Woś et al., 2019). For example, the genus alder (*Alnus*) is considered a metal excluder due to its ability to bind trace elements in the rhizosphere and accumulate them in its roots (Desai et al., 2019; Pourrut et al., 2011; Rosselli et al., 2003). However, birch is a Zn hyperaccumulator, and birch trees on polluted sites may contain up to 4000 mg kg^−1^ Zn in their leaves (Krupnova et al., 2021).

The effect of fire on the concentration and bioaccumulation of PTEs during the initial period of PF ecosystem recovery has been the main focus of investigation thus far (e.g., Abraham et al., 2018; Fernandez-Marcos, 2022; Pereira & Úbeda, 2010; Stankov Jovanovic et al., 2011). However, no data have shown how individual tree species introduced on PF sites have affected the pools of PTEs over the long term. For this reason, we aimed to determine the selected PTE (Al, Cd, Cr, Cu, Mn, Ni, Pb, Zn) concentrations in the soil and their bioaccumulation in the biomass components of different tree species introduced on a large-scale PF site. The following research hypotheses were formulated: (i) the concentration and bioaccumulation of PTEs in the biomass varies significantly between tree species, (ii) the concentration and bioaccumulation of PTEs in the biomass depends more on the tree species than the site (PF vs. undisturbed soils), and (iii) the accumulation of toxic elements (Cd, Cr, Pb) occurs mainly in the roots, with low translocation to the aboveground tree biomass in contrast to microelements (Cu, Mn, Ni, Zn) and beneficial elements (Al), which are characterized by higher translocation factors (TFs).

## Materials and methods

### Study site

The study was located in a reforested site in the Rudziniec Forest District (50.297818 N, 18.421494 E) in southern Poland after a large-scale fire. This region has an average annual precipitation of 578 mm and an annual temperature of 9.7 °C (source, www.tutiempo.net; data for 1990–2022 from meteorological station). Before the fire, the forest mainly contained stands of Scots pine, weakened by long-term emissions of industrial pollutants, including trace elements, from Upper Silesia (Szabla, 1994), which is classed as one of Central Europe’s most polluted regions (Leśniok et al., 2010). The long-term emission of pollutants resulted in the expansion of two species on the forest floor––*Calamagrostis epigejos* (L.) Roth and *Pteridium aquilinum* (L.) Kuhn. Significant amounts of organic matter from the tree species and the slowed rate of decomposition of this organic matter due to the deposition of pollutants led to the formation of large, thick layers of soil organic matter, which were later burned (Szabla, 1994). The fire occurred in August 1992 and covered more than 9000 ha, including 8461 ha of forest land (Szabla, 1994).

### Soil and foliage sampling

The investigated plots were located in Scots pine, silver birch, and black alder stands on a PF site, and the control plots (CPs) were on an undisturbed site (i.e., without fire impact). The age of the PF stands ranged from 26 to 27 years. The sampling plots at the PF site were established on podzols developed from Quaternary quartz sands. The CPs were established in the same managed forests district as the PF plots. The ages of the stands on the CPs were 21 years for pine, 26 years for birch, and 30 years for alder. The soils under the pine and birch stands on the CPs were also podzols. By contrast, those under the alder trees on the undisturbed plots were Luvisols developed from Quaternary loamy sands and sandy loams. The difference in the soil under the alder trees in the undisturbed plots resulted from its strict habitat requirements. This species is not introduced on poor sandy soils, but in disturbed sites, it is introduced as an N-fixer.

In total, 36 research plots were established, representing six replications for each tree species and site combination. Five mineral soil samples were taken from each sample plot (one from the center and one from each of the four corners of the plot), from which a bulk sample was then formed. Samples were taken from 0- to 30-cm deep using a 10-cm-diameter corer.

Samples of the assimilation apparatus (foliage) were collected from treetops with a southwest exposure during the autumn of 2021. Three trees with mean values of diameter at breast height and height were selected from each plot. The collected leaves or needles were pooled to form composite samples. Three soil monoliths (15 × 15 × 30 cm) were collected from under the trees from which the foliage was collected to determine the PTE concentration in the fine roots (diameter < 2 mm).

### Soil and plant analyses

First, the soil samples were dried at 45 °C, then they were sieved (2-mm mesh), and the granulometric composition (texture) was measured using a Fritsch GmbH ANALYSETTE 22 MicroTec plus laser particle sizer. The soil organic carbon (SOC) contents were determined using a LECO TruMac® CNS analyzer. The soil pH in water was determined potentiometrically at a 1:2.5 w/v ratio. The electrical conductivity (EC) was measured using conductometric methods at a temperature of 21 °C and a soil/solution ratio of 1:5. The concentrations of the studied PTEs (Al, Cd, Cr, Cu, Mn, Ni, Pb, Zn) were determined using atomic absorption spectroscopy (iCAP 6000 Series spectrometer, Waltham, MA, USA) after digestion in a mixture of nitric acid (HNO_3_, *d* = 1.40) and 60% perchloric (HClO_4_) acid at a 4:1 ratio.

The fine root samples were stored at 4 °C for less than a week. The roots were then rinsed to remove the soil and a fraction of living roots up to 2 mm thick was selected using a vernier caliper. The roots were then dried at 65 °C and ground to a powder. The foliage samples were also dried at 65 °C and ground to a powder. C and N concentrations in the foliage and fine roots were measured using a LECO TruMac®, and the PTE concentrations were determined using inductively coupled plasma optical emission spectroscopy (iCAP 6000 Series spectrometer) after digestion in a mixture of HNO_3_ (*d* = 1.40) and 60% HClO_4_ acid at a 3:1 ratio.

### Data analysis

The bioaccumulation factor (BAF) and TF were calculated as follows (Bonanno, 2013):

1 $$\textrm{BAF}=\textrm{PTE}\ \textrm{in}\ \textrm{biomass}\ \textrm{component}/\textrm{PTE}\ \textrm{in}\ \textrm{soil}$$

2 $$\textrm{TF}=\textrm{PTE}\ \textrm{in}\ \textrm{foliage}/\textrm{PTE}\ \textrm{in}\ \textrm{fine}\ \textrm{roots}$$

The effects of the investigated tree species and the site on the basic soil characteristics and PTE concentrations were analyzed using a two-way analysis of variance (ANOVA) at *p* < 0.05. If overall significant effects of the site, species, or their interaction were observed using the ANOVA, Tukey’s honest significant difference test was used to compare the respective means. The correlations between the PTE concentrations in the soil, fine roots, and foliage were described using a Pearson’s correlation matrix at *p* < 0.05. The datasets were analyzed using the Statistica 13.3 software.

## Results

### Soil characteristics

The PF soils contained more sand and less silt than the CP soils. The pH was similar at both sites, but the EC was higher at the CP than at the PF site. The SOC was also higher at the CP site than at the PF site (Table S1).

The sites, tree species, and their interactions significantly affected the soil concentrations of the studied PTEs. The PTE contents in the soils were higher at the CP site than at the PF site. The Al, Cr, Mn, Pb, and Ni concentrations were similar in the soils under birch and alder but were lower under pine. The lowest Cd concentration was found in the soil under pine, and the highest was under birch. The highest concentration of Cr was in the soil under birch and was statistically lower in the soils under pine and alder. The highest Zn content in the soil was under alder, which was statistically higher than the soil Zn concentration under pine and birch (Table 1).

**Table 1:** Potentially toxic element (PTE) concentrations in the studied soils at the post-fire (PF) and control plot (CP) sites

Effect		Al	Cd	Cr	Cu	Mn	Ni	Pb	Zn
		[mg kg^−1^]							
Site		*^1^	*	*	*	*	*	*	*
PF		2677.73 ± 153.62^a 2^	0.35 ± 0.05^a^	3.91 ± 0.54^a^	1.89 ± 0.16^a^	16.36 ± 2.18^a^	1.21 ± 0.09^a^	17.18 ± 1.24^a^	12.25 ± 2.89^a^
CP		7701.31 ± 1033.61^b^	0.60 ± 0.09^b^	9.20 ± 0.69^b^	3.73 ± 0.46^b^	41.91 ± 7.64^b^	4.07 ± 0.66^b^	31.66 ± 3.24^b^	32.13 ± 6.05^b^
Species		*	*	*	*	*	*	*	*
Pine		2861.68 ± 472.46^a^	0.30 ± 0.03^a^	5.44 ± 0.30^a^	1.70 ± 0.24^a^	11.48 ± 0.97^a^	1.12 ± 0.18^a^	18.16 ± 1.93^a^	8.47 ± 1.13^a^
Birch		5369.13 ± 1041.10^b^	0.62 ± 0.10^b^	8.71 ± 1.06^b^	3.45 ± 0.55^b^	39.18 ± 8.51^b^	2.95 ± 0.57^b^	27.03 ± 3.76^b^	18.63 ± 3.29^a^
Alder		6740.10 ± 1363.87^b^	0.48 ± 0.11^ab^	5.16 ± 1.29^a^	3.09 ± 0.46^b^	33.98 ± 7.85^b^	3.52 ± 0.87^b^	26.56 ± 4.12^b^	36.37 ± 8.14^b^
Species × site		*	*	*	*	*	*	*	*
PF	Pine	2908.04 ± 301.01^a^	0.34 ± 0.04^ab^	4.96 ± 0.24^b^	2.00 ± 0.31^a^	13.27 ± 1.04^a^	1.31 ± 0.09^b^	19.23 ± 1.69^a^	9.05 ± 1.00^a^
	Birch	2765.79 ± 205.88^a^	0.54 ± 0.11^b^	5.87 ± 0.30^c^	2.07 ± 0.36^a^	25.81 ± 4.41^b^	1.51 ± 0.12^b^	18.22 ± 2.01^a^	10.98 ± 2.37^a^
	Alder	2359.35 ± 274.39^a^	0.17 ± 0.03^a^	0.90 ± 0.19^a^	1.62 ± 0.17^a^	9.99 ± 0.47^a^	0.80 ± 0.08^a^	14.10 ± 2.44^a^	16.73 ± 8.50^a^
CP	Pine	2806.05 ± 1044.21^a^	0.26 ± 0.03^a^	6.01 ± 0.50^a^	1.34 ± 0.34^a^	9.34 ± 1.20^a^	0.88 ± 0.37^a^	16.87 ± 3.94^a^	7.78 ± 2.31^a^
	Birch	8493.13 ± 1193.64^b^	0.72 ± 0.17^b^	12.12 ± 0.83^c^	5.12 ± 0.45^b^	55.23 ± 15.85^b^	4.68 ± 0.63^b^	37.61 ± 4.51^b^	27.82 ± 3.54^b^
	Alder	11,120.85 ± 657.99^b^	0.80 ± 0.12^b^	9.42 ± 0.22^b^	4.57 ± 0.21^b^	57.96 ± 6.37^b^	6.23 ± 0.64^b^	39.03 ± 2.58^b^	56.01 ± 8.05^b^

^1^Results of two-way ANOVA for the effect of site, species, and its interaction, i.e., (*), significant; (-), differences are not significant (*p* < 0.05)

^2^Mean ± SE; different letters indicate significant differences within columns

### PTE concentrations in tree biomass components

**Fig. 1:**
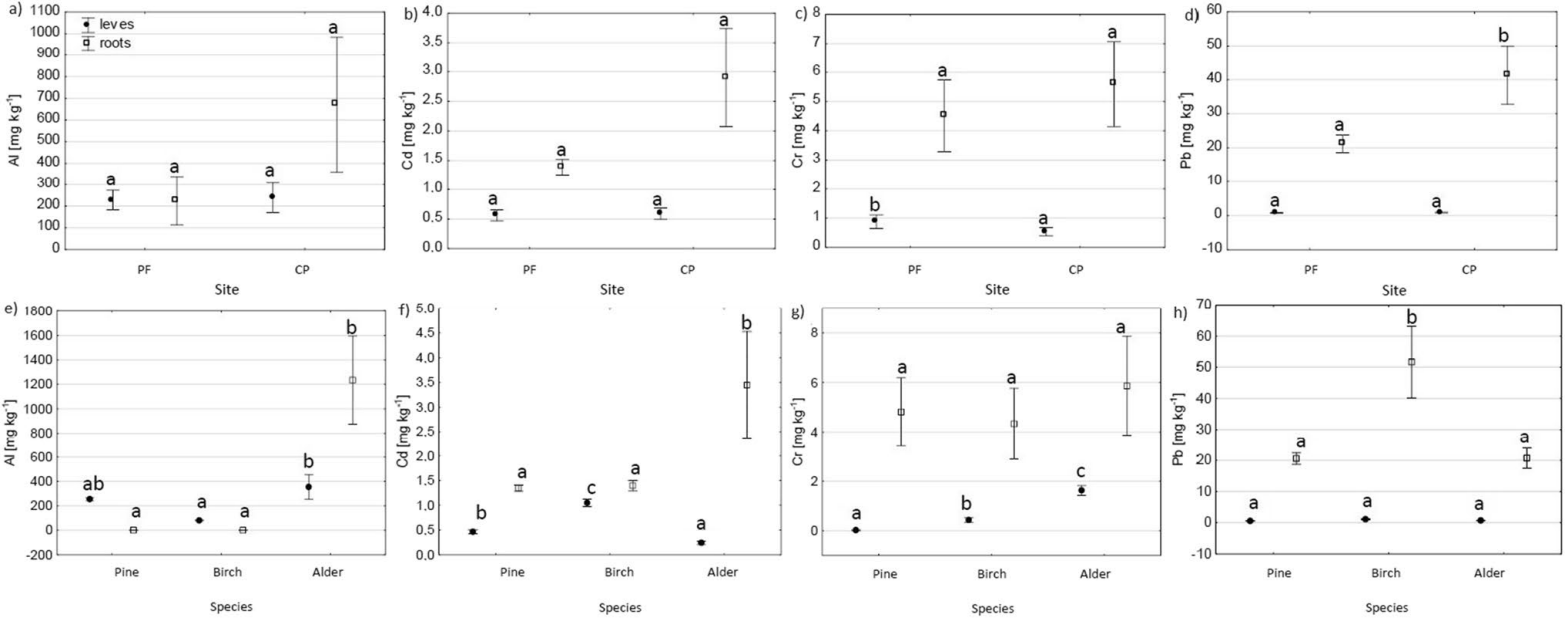
Effect of site (**a**–**d**) and tree species (**e**–**h**) on Al, Cd, Cr, and Pb concentrations in the fine roots and foliage. The different letters (a, b, and c) on the plots indicate significant differences (at *p* < 0.05) in the PTE concentrations in roots and leaves, separately

**Fig. 2:**
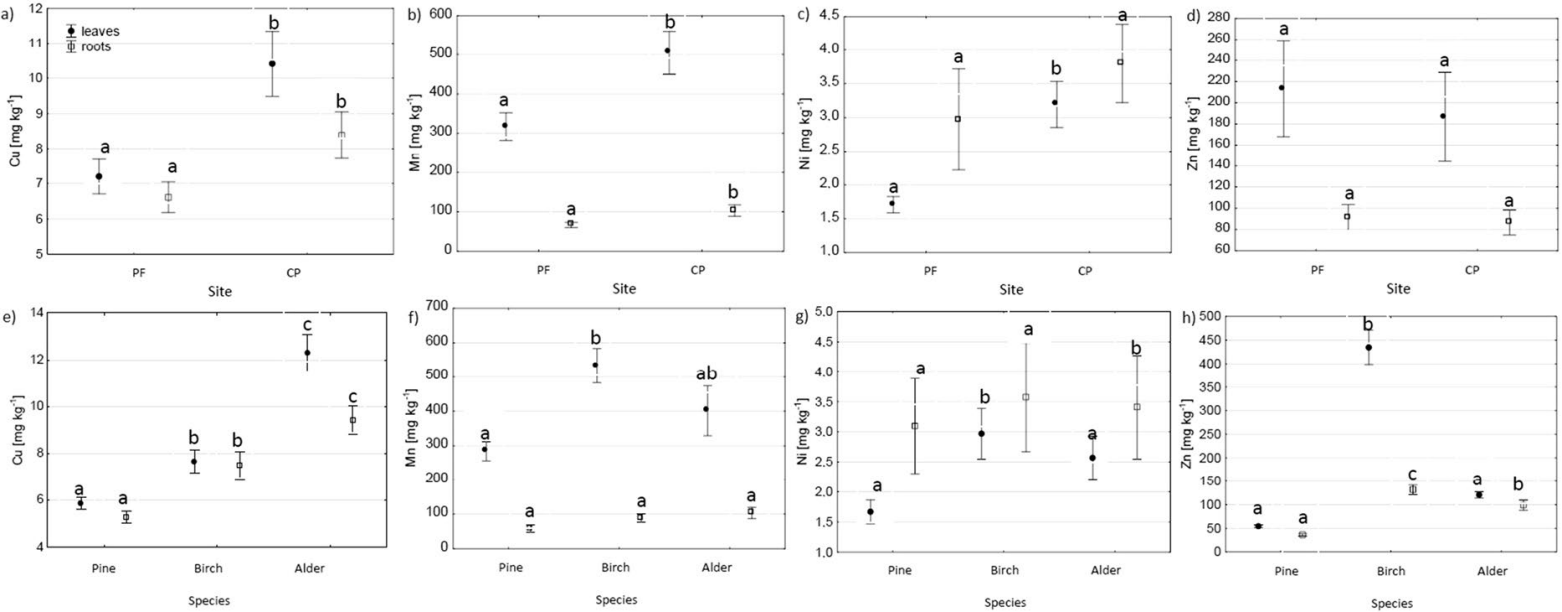
Site (**a** and **b**) and tree species (**e**–**h**) effect on microelement (Cu, Mn, Ni, and Zn) concentrations in the fine roots and foliage. The different letters (a, b, and c) on the plots indicate significant differences (at *p* < 0.05) in the PTE concentrations in roots and leaves, separately

Upon comparing the sites (PF vs. CP), we found that the highest differences in PTE concentrations in the fine roots were those of Cu, Mn, and Pb. Higher concentrations of these elements were found at the CP site than at the PF site (Figs. 1 and 2).

Upon comparing the tested tree species, we observed differences in the PTE concentrations in the fine roots in Al, Cd, Cu, Pb, Zn, Al, Cd, and Cu (Figs. 1 and 2). The Al and Cd were higher in the fine roots of alder than in the roots of birch and pine. The Pb concentrations were higher in the birch roots than in the pine and alder roots (Fig. 1). The Cu concentrations were highest in the alder roots, lower in the birch roots, and lowest in the pine roots. The Zn concentrations were also highest in the birch roots, lower in the alder roots, and lowest in the pine roots (Fig. 2). The tree species × site interaction did not significantly affect the PTE concentrations in the fine roots (Table S2).

Upon comparing the sites (PF vs. CP), we observed differences in the PTE concentrations in the tree foliage in Cr, Cu, Mn, and Ni (Figs. 1 and 2). The Cr concentrations in the tree foliage were higher at the PF site than at the CP site (Fig. 1). Contrastingly, the foliage concentrations of Cu, Mn, and Ni were higher at the CP site than at the PF site (Fig. 2).

Between-species differences in the foliage were observed for all studied PTE concentrations (Figs. 1 and 2). The Al concentrations were highest in the alder and lowest in the birch foliage. The Cd concentrations were highest in the birch leaves and lowest in the alder leaves (Fig. 1). Otherwise, the lowest values for all tested elements were found in the pine foliage. The Cr and Cu concentrations were highest in the alder leaves and lowest in the pine needles. The Mn concentrations were highest in the birch and lowest in the pine foliage. The Pb and Zn concentrations were highest in the birch and lowest in the pine foliage (Figs. 1 and 2).

The tree species × site interaction affected only the Ni concentrations. The Ni concentrations at the CP site were similar in the birch and alder leaves and lower in the pine needles. No differences were found in the Ni concentrations in the foliage of the studied tree species at the PF site (Table S3).

### PTE bioaccumulation

**Fig. 3:**
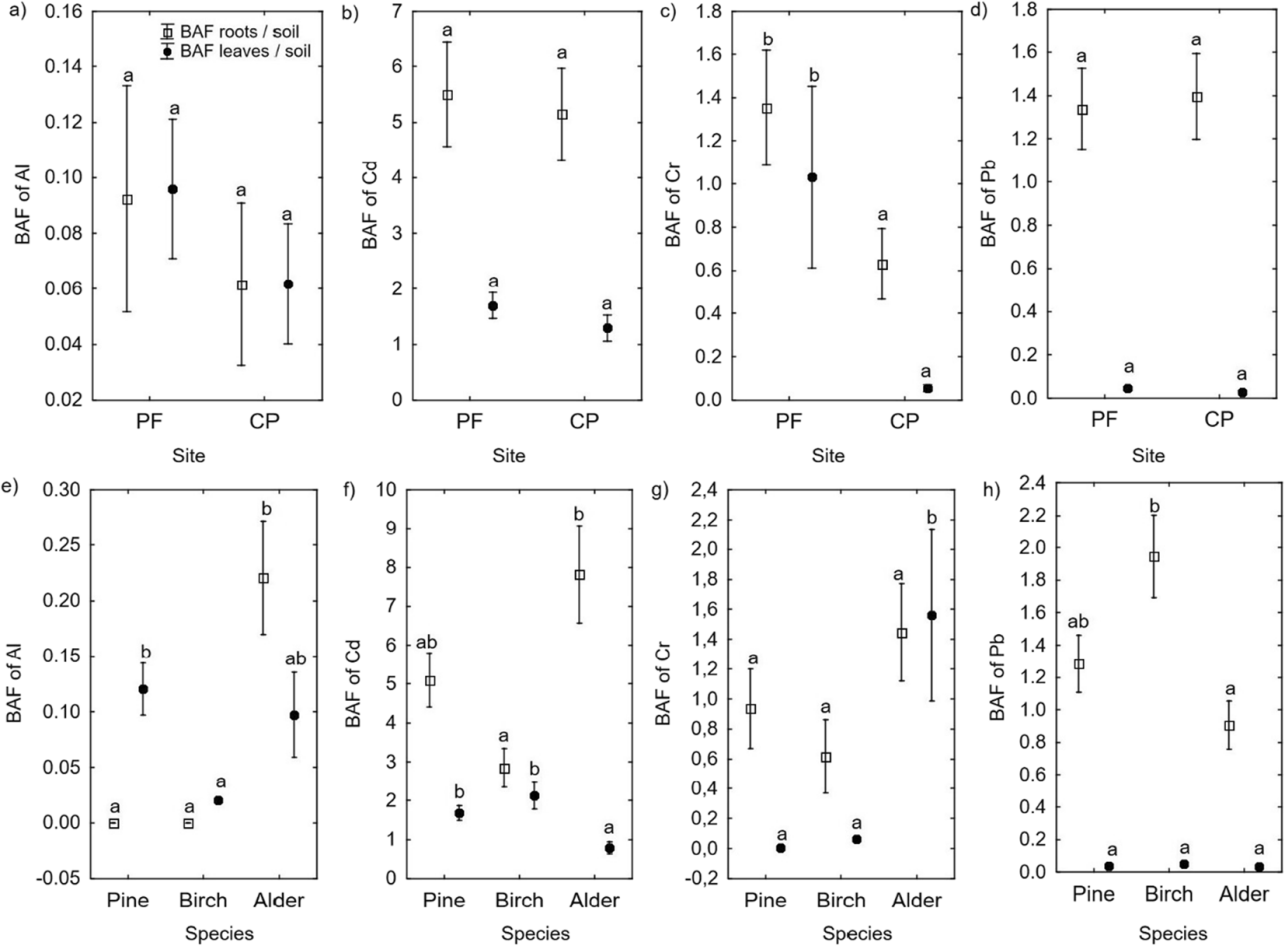
Site (**a**–**d**) and tree species (**e**–**h**) effect on the Al, Cd, Cr, and Pb BAFs. The different letters (a and b) on the plots indicate significant differences (at *p* < 0.05) in the BAFs for the roots/soil and leaves/soil groups, separately

**Fig. 4:**
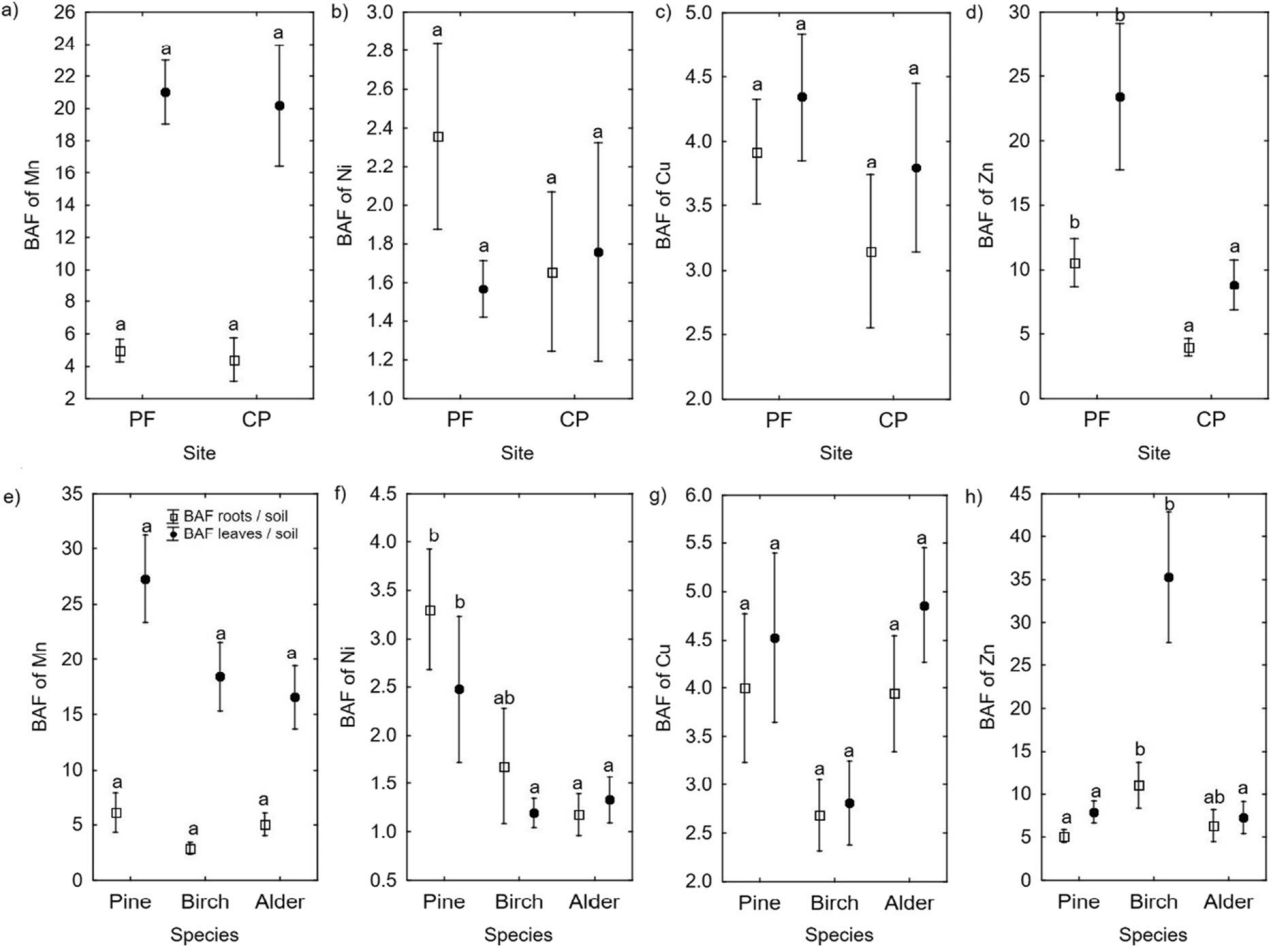
Site (**a** and **b**) and tree species (**e**–**h**) effect of tree species on the BAFs of the studied microelements (Mn, Ni, Cu, and Zn). The different letters (a and b) indicate significant differences (at *p* < 0.05) in the BAFs for the roots/soil and leaves/soil groups, separately

The site effect on the BAF was different only for Cr and Zn. The roots/soil and leaves/soil BAFs for Cr and Zn were higher at the PF site than at the CP site (Figs. 3 and 4).

Regarding the effects of tree species on the BAF, a higher roots/soil BAF for Al was found in alder than in pine and birch. The highest leaves/soil BAF for Al was in pine, and the lowest was in birch. Similarly, the highest roots/soil BAF for Cd was in alder, and the lowest was in birch. The leaves/soil BAF for Cd was higher in pine and birch than in alder. The roots/soil BAF for Cr was similar in all three tree species, but the leaves/soil BAF for Cr was higher in alder than in pine and birch. The roots/soil BAF for Pb was higher in birch than in alder. However, the roots/soil BAF for Pb was similar in all three tree species (Fig. 3). The highest roots/soil BAF for Ni was in pine, and the lowest was in alder. The highest leaves/soil BAF for Ni was also in pine, with lower values in both birch and alder. The roots/soil BAF for Zn was highest in birch and lowest in pine. The leaves/soil BAF for Zn was higher in birch than in pine or alder. No differences in the BAFs for Cu or Mn were found between the three tree species (Fig. 4). The tree species × site interaction effect was significant in the roots/soil BAFs for Cu and Mn (Table S4) and the leaves/soil BAFs for Al, Cu, Mn, and Ni (Table S5).

### PTE translocation

The TF was higher at the PF site than at the CP site only for Cr. For the remaining trace elements, no differences were found between the sites (Table 2).

**Table 2:** Translocation factors (TFs) of the trace elements in the tree species biomass in the PF and CP sites

		TF of Al	TF of Cd	TF of Cr	TF of Cu	TF of Mn	TF of Ni	TF of Pb	TF of Zn
Site		-^1^	-	*	-	-	-	-	-
PF		1463.05 ± 449.42^a 2^	0.46 ± 0.08^a^	0.92 ± 0.39^b^	1.10 ± 0.05^a^	5.40 ± 0.62^a^	1.19 ± 0.22^a^	0.04 ± 0.01^a^	2.12 ± 0.26^a^
CP		1574.36 ± 523.53^a^	0.39 ± 0.09^a^	0.13 ± 0.05^a^	1.27 ± 0.09^a^	5.93 ± 0.90^a^	1.00 ± 0.10^a^	0.03 ± 0.00^a^	2.21 ± 0.41^a^
Species		*	*	*	*	*-	*-	*-	*
Pine		4005.96 ± 458.84^c^	0.36 ± 0.04^a^	0.01 ± 0.00^a^	1.13 ± 0.07^ab^	6.02 ± 0.86^a^	0.83 ± 0.16^a^	0.03 ± 0.00^a^	1.57 ± 0.14^a^
Birch		677.46 ± 97.17^b^	0.81 ± 0.10^b^	0.17 ± 0.04^b^	1.05 ± 0.06^a^	7.09 ± 1.13^a^	1.08 ± 0.14^a^	0.03 ± 0.00^a^	3.51 ± 0.44^b^
Alder		0.58 ± 0.20^a^	0.15 ± 0.05^a^	1.40 ± 0.54^c^	1.35 ± 0.10^b^	3.99 ± 0.49^a^	1.36 ± 0.29^a^	0.05 ± 0.01^a^	1.46 ± 0.26^a^
Species × site		-	-	*	-	-	-	-	-
PF	Pine	3661.57 ± 729.23^c^	0.34 ± 0.04^a^	0.01 ± 0.00^a^	1.11 ± 0.11^a^	5.77 ± 0.78^ab^	0.56 ± 0.21^a^	0.04 ± 0.00^a^	1.36 ± 0.10^a^
	Birch	726.70 ± 160.09^b^	0.83 ± 0.12^b^	0.19 ± 0.06^b^	1.05 ± 0.05^a^	7.27 ± 1.09^b^	0.98 ± 0.21^ab^	0.03 ± 0.00^a^	3.25 ± 0.23^b^
	Alder	0.88 ± 0.35^a^	0.22 ± 0.09^a^	2.58 ± 0.85^c^	1.14 ± 0.07^a^	3.15 ± 0.59^a^	2.02 ± 0.43^b^	0.06 ± 0.02^b^	1.75 ± 0.49^a^
CP	Pine	4419.22 ± 531.01^c^	0.39 ± 0.08^a^	0.01 ± 0.00^a^	1.16 ± 0.06^ab^	6.32 ± 1.77^a^	1.14 ± 0.17^a^	0.03 ± 0.01^a^	1.83 ± 0.25^a^
	Birch	618.38 ± 109.05^b^	0.78 ± 0.17^b^	0.14 ± 0.04^b^	1.04 ± 0.11^a^	6.87 ± 2.29^a^	1.21 ± 0.19^a^	0.02 ± 0.01^a^	3.82 ± 0.98^b^
	Alder	0.28 ± 0.16^a^	0.07 ± 0.02^a^	0.23 ± 0.12^b^	1.55 ± 0.16^b^	4.82 ± 0.66^a^	0.70 ± 0.12^a^	0.03 ± 0.01^a^	1.18 ± 0.12^a^

^1^Results of two-way ANOVA for the effect of site, species, and its interaction, i.e., (*), significant; (-), differences are not significant (*p* < 0.05)

^2^Mean ± SE; different letters indicate significant differences within columns

Different translocation patterns were found among the various species. The TF for Al was the highest in pine and the lowest in alder. By contrast, the TF for Cr was the highest in alder and the lowest in pine. The TFs for Cd and Zn were higher in birch than in the other two species. The TF for Cu was the highest in alder and the lowest in birch. The TFs for Mn, Ni, and Pb were similar across the three tree species (Table 2).

### Correlations in the studied plant–soil system

The concentrations of the studied PTEs in the soil correlated negatively with sand and positively with silt, clay, and SOC content; pH (except for Cr); and EC (except for Cd, Cr, and Mn) (Table 3).

**Table 3:** Correlation coefficients (*r*) between the soil pH, texture, electrical conductivity (EC), soil organic carbon (SOC), and PTE concentrations in the studied soils

PTE concentration in soil	Soil properties					
	Sand	Silt	Clay	pH	EC	SOC
Al	−0.91*^1^	0.91*	0.62*	0.57*	0.59*	0.59*
Cd	−0.63*	0.65*	0.36*	0.51*	-	0.63*
Cr	−0.72*	0.72*	0.53*	-^2^	-	0.45*
Cu	−0.73*	0.77*	0.45*	0.45*	0.53*	0.77*
Mn	−0.72*	0.72*	0.52*	0.55*	-	0.39*
Ni	−0.90*	0.90*	0.62*	0.60*	0.53*	0.57*
Pb	−0.73*	0.77*	0.43*	0.40*	0.55*	0.77*
Zn	−0.76*	0.76*	0.45*	0.57*	0.58*	0.59*

^1^(*), significant at *p* < 0.05

^2^(-), not significant

Apart from Cr and Zn, the PTE concentrations in the roots correlated positively with their concentrations in the soil. The Al, Cd, Cr, Cu, Mn, and Ni in the roots correlated positively with silt and negatively with sand content. Only the Cd concentration in the roots correlated positively with clay content. The Al, Cd, Cr, Ni, and Zn concentrations correlated positively with pH. The Al and Cu concentrations correlated positively with EC. Positive correlations were also found between Cu and Pb in the roots and SOC content (Table 4).

**Table 4:** Correlation coefficients (*r*) between the soil properties and the PTE concentrations in the fine roots

PTE concentration in roots	Soil properties													
	Al	Cd	Cr	Cu	Mn	Ni	Pb	Zn	Sand	Silt	Clay	pH	EC^1^	SOC^2^
Al	0.47*^3^	0.34*	-	-	0.42*	0.44*	0.44*	0.79*	−0.48*	0.49*	-	0.35*	0.39*	-
Cd	0.54*	0.46*	-	0.37*	0.37*	0.59*	0.40*	0.55*	−0.54*	0.53*	0.44*	0.46*	-	-
Cr	0.38*	-	-	-	-	0.40*	-	-	−0.39*	0.40*	-	0.34*	-	-
Cu	0.50*	-	-	0.57*	0.45*	0.48*	0.56*	0.55*	−0.47*	0.47*	-	-	0.55*	0.58*
Mn	0.42*	0.39*	-	0.35*	0.62*	0.43*	0.35*	0.54*	−0.45*	0.43*	-	-	-	-
Ni	0.43*	-	0.36*	0.40*	-	0.45*	0.35*	-	−0.39*	0.39*	-	0.37*	-	-
Pb	-^4^	-	0.51*	0.53*	-	-	0.54*	-	-	-	-	-	-	0.41*
Zn	-	0.39*	-	0.37*	0.44*	-	-	-	-	-	-	0.38*	-	-

^1^*EC* electrical conductivity

^2^*SOC* soil organic carbon

^3^(*), significant at *p* < 0.05

^4^(-), not significant

The Cr concentration in the foliage correlated negatively with the Cr content in the soil. There was a positive correlation between the foliage and soil concentrations of Cu, Mn, Ni, and Pb. The concentrations of micronutrients (Cu, Mn, Ni) in the tree foliage correlated positively with silt and clay and negatively with sand content. A positive correlation was also found between the Cu and Ni concentrations in the foliage and pH and EC. The Cu, Ni, and Pb concentrations also correlated with the SOC content (Table 5).

**Table 5:** Pearson correlation coefficients (*r*) between the PTE concentrations in the tree foliage and the soil properties

PTE concentration in foliage	Soil properties													
	Al	Cd	Cr	Cu	Mn	Ni	Pb	Zn	Sand	Silt	Clay	pH	EC^1^	SOC^3^
Al	-^3^	-	-	-	-	-	-	-	-	-	-	-	-	-
Cd	-	-	0.44*	-	-	-	-	-	-	-	-	-	-	-
Cr	-	-	−0.40*	-	-	-	-	-	-	-	-	-	-	-
Cu	0.71*^4^	0.38*	-	0.56*	0.52*	0.69*	0.56*	0.72*	−0.81*	0.78*	0.48*	0.38*	0.65*	0.48*
Mn	0.53*	0.56*	0.65*	0.51*	0.65*	0.55*	0.55*	0.53*	−0.55*	0.53*	0.35*	-	-	-
Ni	0.68*	0.44*	0.72*	0.69*	0.71*	0.71*	0.66*	0.53*	−0.69*	0.68*	0.49*	0.37*	0.50*	0.45*
Pb	-	0.38*	0.47*	0.46*	-	0.35*	0.37*	-	-	-	-	-	-	0.38*
Zn	-	0.37*	0.36*	-	-	-	-	-	-	-	-	-	-	-

^1^*EC* electrical conductivity

^2^*SOC* soil organic carbon

^3^(-), insignificant at *p* < 0.05

^4^(*), significant at *p* < 0.05

## Discussion

The concentrations of the studied PTEs in the soils at the PF and CP sites (Table 1) were low and did not exceed the values given for unpolluted sites (Kabata-Pendias, 2001; Terzano et al., 2021). These results confirm that the mobilization of PTEs by fire and their associated risks are low in unpolluted sites (Fernandez-Marcos, 2022). Higher PTE concentrations were found in the soils at the CP site than at the PF site (Table 1), possibly resulting from the higher silt fraction and SOC content in the former (Table S1) (Kabała et al., 2020; Kirchmann & Eriksson, 2011), as confirmed by the correlation analysis (Table 3). Moreover, during a fire, PTEs are released due to biomass and dead organic matter combustion and the transformation of minerals containing these elements (Fernandez-Marcos, 2022). These elements are then accumulated in ash, which can be dispersed by wind or surface runoff, or leached into deeper soil horizons (Bodí et al., 2014; Campos et al., 2016). Due to these processes, the concentrations of trace elements in soils may decrease with time after a fire (Campos et al., 2016). Despite lower Cr concentrations in the soils (Table 1), the foliage of the trees at the PF site was characterized by higher Cr concentrations compared to the trees at the CP site (Fig. 1). In addition, the roots/soil and leaves/soil BAFs (Fig. 3) and TF for Cr (Table 2) were higher at the PF site than the CP site. Fire drives the oxidation of poorly soluble Cr(III) cations substituted within Fe oxides, transforming them into highly soluble oxyanions of Cr(VI) (Burton et al., 2019; Panichev et al., 2008). The results indicate that the fire signature for Cr may have persisted, even 30 years after the fire.

The BAFs, TFs, and concentrations of PTEs in the fine roots and foliage showed different patterns in the three tree species. These patterns depended more on the species than the sites. For this reason, it can be assumed that these tree species would be characterized by a similar pattern of PTE distributions in the biomass in other, more-polluted, habitats. This feature could be used in the remediation and phytostabilization of other contaminated soils. However, we do not know this for sure, and therefore, verification studies will be needed. Alder was characterized by high Al concentrations in the fine roots (Fig. 1) and the lowest Al TF (Table 2). The high Al concentration in the alder roots may be due to several reasons. During N-fixation and the nitrification process, oxidation reactions produce HNO_3_, which then dissociates into separated nitrate (NO^3−^) and hydrogen (H^+^) ions, increasing the soil acidity (Cole et al., 1990). High Al concentrations in the roots likely ameliorate H^+^ toxicity (Broadley et al., 2012), and Al also alleviates the toxicity caused by other elements, such as P and Fe (Broadley et al., 2012). Phosphorus (P) toxicity is relatively uncommon, occurring mainly in tree species adapted to habitats poor in P after its content suddenly increases, such as in the case of fertilization (Lambers, 2022). Because P is needed in the process of N-fixation, N-fixers require more P than non-fixers (Ardanuy et al., 2021). For example, Chodak et al. (2021) reported greater P storage in the microbial biomass and higher phosphatase activity in post-mining soils under N-fixing trees (black locust and black alder) than under non-N-fixing trees (Scots pine and silver birch) in afforested post-mining areas in Poland. Alder naturally occurs in humid habitats with soils characterized by a high Fe content, and it can tolerate soil Fe concentrations that are toxic to many plants (Funk, 1990). The other tree species in this study––pine and birch––were characterized by lower Al concentrations in their fine roots (Fig. 1), but with higher Al TFs than in alder (Table 2). As mentioned, the role of Al in plants is not fully understood; although, in small amounts, it may improve plant growth and resistance to herbivory (Bojórquez-Quintal et al., 2017; Pilon-Smits et al., 2009). This beneficial effect has been mainly observed in species adapted to acidic soils, such as pine and birch (Bojórquez-Quintal et al., 2017).

The Cd concentrations in the foliage (Fig. 1) and Cd TF (Table 2) increased from alder to pine to birch at both studied sites. The highest Cd concentrations found in the birch leaves confirmed the ability of this species to accumulate Cd (Rosselli et al., 2003). Similarly, higher Cd concentrations have been found in birch leaves than in pine needles in forest ecosystems due to pollution from Pb–Zn mining and ore-processing plants in Poland (Pająk et al., 2017). Higher Cd concentrations have also been found in birch leaves than in alder leaves at sites reclaimed after opencast coal mining in the UK (Desai et al., 2019). In contrast to Cd concentrations in the leaves, the highest Cd concentration and Cd BAF occurred in the fine roots of the alder (Fig. 1). Alder is known for high Cd concentrations in its roots and low Cd concentrations in its aboveground biomass (Rosselli et al., 2003).

The Cr concentrations in the foliage and the Cr TFs for the studied tree species increased from pine to birch to alder (Fig. 1). Plants can take up Cr as Cr(III) or Cr(VI) cations (Shahid et al., 2017). As mentioned, Cr(III) ions are poorly soluble and so are available for plants, their uptake being a passive process that does not require energy (Shahid et al., 2017). Unlike Cr(III), Cr(VI) uptake is active, occurring mainly through the sulfate or phosphate transporter due to the structural similarities of Cr, S, and P (de Oliveira et al., 2016; Saleem et al., 2022; Shahid et al., 2017). For this reason, species characterized by high S and P concentrations in their organs may also be characterized by higher concentrations of Cr (Ali et al., 2023). Birch is a more demanding species than pine and usually contains higher amounts of S and P in its organs (Heinsdorf, 1999; Kuznetsova et al., 2011). Accordingly, higher Cr concentrations have been found in birch leaves than in pine needles in forest ecosystems under the influence of pollution from Pb–Zn ore mining in Poland (Pająk et al., 2017). Furthermore, N-fixing species, such as alders, are in high demand for their S and P contents because these elements are necessary for N-fixation (Ardanuy et al., 2021; Varin et al., 2010). Although Cr does not play any significant physiological role in plants (Shahid et al., 2017), when present in low concentrations in the soil, it may support the formation of nodules in N-fixers (Saleem et al., 2022).

The higher microelement concentrations in birch and alder than in pine, such as the higher Ni and Cu in birch and alder leaves and Mn in birch leaves than in pine needles (Fig. 2), may result from the different habitat requirements of deciduous species (Ellenberg, 2009). Tree species with greater habitat and nutrient requirements typically contain more nutrients in their organs (Poorter & de Jong, 1999; Wright & Westoby, 2003). For example, in reclaimed sites after oil-shale mining in Estonia, alder and birch required more nutrients for growth than pine (Kuznetsova et al., 2011). Higher microelement concentrations in alder and birch leaves may also partially result from the lower transpiration rate in pine (Gagnon et al., 2020). The higher microelement contents in the birch and alder foliage indicate that these species stimulated the biogeochemical cycling of these elements during ecosystem recovery more than pine. By contrast, pine uses micronutrients more efficiently (Kuznetsova et al., 2011).

Birch had the highest Pb (Fig. 1) and Zn (Fig. 2) concentrations in the fine roots and foliage among the studied species. However, studies have yet to determine that birch can accumulate more Pb than the other species. Alder leaves have been found to contain more Pb than birch leaves at sites reclaimed after coal mining in the UK (Desai et al., 2019), and similar Pb contents have been found in the foliage of birch and pine trees growing near a former Zn and Pb smelter in Poland (Pająk et al., 2017; Zakrzewska & Klimek, 2018). However, the highest Zn concentration found in the birch organs was consistent with data from the literature, indicating that this species is among the plants that hyperaccumulate Zn (Dmuchowski et al., 2013). The Zn concentration in the birch foliage at the CP site did not exceed those found in unpolluted sites (up to 400 mg kg^−1^) (Dmuchowski et al., 2013), and it only slightly exceeded the concentration at the PF site.

Whereas PTEs are thought to accumulate mainly in roots, with limited ability for the elements to translocate to the aboveground biomass (TF < 1 classified as metal-excluder species) (Antoniadis et al., 2021; Bonanno, 2013; Woś et al., 2019), our study can only partially confirm this. Concentrations of toxic elements were higher in the roots than in the leaves (Fig. 1). Concentrations of the other microelements (Mn, Zn, and Cu) in the foliage were similar or higher than in the roots (Fig. 2). Considered a beneficial element, Al had higher concentrations in the roots than in the foliage (Fig. 1) and a low TF (0.58) in alder (Table 2). In birch and pine, an opposite phenomenon occurred––higher Al concentrations occurred in the leaves than in the roots (Fig. 1), and the TFs were very high (4005.96 and 677.46, respectively) (Table 2). However, despite the high TF, Al was poorly taken up by these tree species, as indicated by Al having the lowest BAF (< 0.25) compared to those of the other analyzed elements (Figs. 3 and 4). Similarly, in previous studies on the phytomeliorative role of the three wetland species (*Phragmites australis*, *Typha domingensis*, and *Arundo donax*), Al was the least absorbed element by the plants compared to Hg, As, Cd, Cu, Cr, Mn, Pb, Zn, and Ni (Bonanno, 2013).

## Conclusion

There was no direct threat from the tested PTEs at a reforested site 30 years after a fire. However, despite the lower Cr concentrations in the soils at the post-fire sites (PF) than at the control sites (CP), Cr was characterized by higher mobility in the plant–soil system at the PF site. The concentrations of the tested PTEs in the fine roots and foliage and their BAFs and TFs showed different patterns across the three tree species that depended more on the species than the site. This suggests that similar patterns might also occur in other polluted soils. For this reason, different species-dependent bioaccumulation patterns could also be used in the phytostabilization and remediation of other polluted sites that are under industrial pressure.

## Supplementary information

ESM 1 (DOCX 32 kb)

## Data Availability

The datasets are available upon reasonable request from the corresponding author.
