# Supplementary material for: How tree species have modified the potentially toxic elements distributed in the developed soil–plant system in a post-fire site in highly industrialized region
Source: Environ Monit Assess. 2024 Aug 3;196(9):780. doi: 10.1007/s10661-024-12933-3 (PMC11297815; doi:10.1007/s10661-024-12933-3)
Supplement: Supplementary file 1 — (DOCX 32 kb) [file 10661_2024_12933_MOESM1_ESM.docx]

Table S1. Basic soil parameters for post-fire (PF) and control plots (CP)

| Effect | | Sand (2 – 0.05 mm) | Silt (0.05 – 0.002 mm) | Clay (< 0.002 mm) | pH | EC ^1^ | SOC ^2^ |
| --- | --- | --- | --- | --- | --- | --- | --- |
|  |  | [%] | | |  | [μS cm^−1^] | [%] |
| Site | | *^1^ | * | - | - | * | * |
| PF | | 89±0^b 2^ | 10±1^a^ | 2±0^a^ | 4.3±0.1^a^ | 31.17±3.70^a^ | 2.23±0.25^a^ |
| CP | | 73±3^a^ | 24±2^b^ | 3±0^a^ | 4.3±0.1^a^ | 57.31±2.64^b^ | 3.44±0.38^b^ |
| Species | | * | * | - | * | * | * |
| Pine | | 87±1^c^ | 12±1^a^ | 2±0^a^ | 4.3±0.1^b^ | 35.73±5.47^a^ | 1.83±0.18^a^ |
| Birch | | 83±3^b^ | 15±2^a^ | 2±0^a^ | 4.6±0.1^c^ | 38.09±6.29^a^ | 3.23±0.54^b^ |
| Alder | | 76±4^a^ | 22±4^b^ | 2±0^a^ | 4.0±0.1^a^ | 55.50±3.13^b^ | 3.30±0.33^b^ |
| Species × Site | | * | * | - | * | * | * |
| PF | Pine | 88±1^a^ | 12±1^a^ | 2±0^a^ | 4.5±0.0^b^ | 22.17±1.25^a^ | 1.67±0.09^a^ |
|  | Birch | 89±1^a^ | 9±1^a^ | 2±0^a^ | 4.8±0.1^b^ | 21.00±2.03^a^ | 2.08±0.41^a^ |
|  | Alder | 90±0^a^ | 9±0^a^ | 1±0^a^ | 3.6±0.1^a^ | 50.33±4.85^b^ | 2.96±0.53^a^ |
| CP | Pine | 85±1^c^ | 13±1^a^ | 2±0^a^ | 4.1±0.0^a^ | 52.00±6.41^a^ | 2.03±0.38^a^ |
|  | Birch | 76±3^b^ | 22±3^b^ | 2±0^a^ | 4.4±0.1^a^ | 58.60±4.28^a^ | 4.62±0.70^b^ |
|  | Alder | 62±1^a^ | 34±1^c^ | 4±0^a^ | 4.3±0.1^a^ | 60.67±2.99^a^ | 3.63±0.37^ab^ |
| ^1^ EC – electrical conductivity  ^2^ SOC – soil organic carbon  ^3^ Results of two-way ANOVA for the effect of site, species, and its interaction, i.e.: * – significant; - – differences not significant (p < 0.05)  ^4^ Mean ± SE; different letters indicate significant differences within columns | | | | | | | |

Table S2. Interaction tree species × site effect on PTEs concentrations in the fine roots of the studied tree species

| Effect | | Al | Cd | Cr | Cu | Mn | Ni | Pb | Zn |
| --- | --- | --- | --- | --- | --- | --- | --- | --- | --- |
|  |  | [mg kg^-1^] | | | | | | | |
| Species × Site | | - | - | - | - | - | - | - | - |
| PF | Pine | 0.09±0.02^a^ | 1.37±0.05^a^ | 7.05±2.15^a^ | 4.88±0.21^a^ | 42.99±7.64^a^ | 4.35±1.24^a^ | 18.99±1.46^a^ | 40.55±3.08^a^ |
|  | Birch | 0.13±0.03^a^ | 1.36±0.18^a^ | 5.35±2.62^a^ | 6.20±0.29^a^ | 77.87±12.47^a^ | 3.56±1.70^a^ | 32.44±4.26^b^ | 147.96±11.23^c^ |
|  | Alder | 678.12±262.79^b^ | 1.43±0.37^a^ | 1.14±0.25^a^ | 8.75±0.51^b^ | 78.68±9.75^a^ | 1.00±0.23^a^ | 12.16±2.55^a^ | 84.82±14.69^b^ |
| CP | Pine | 0.06±0.01^a^ | 1.32±0.13^a^ | 2.11±0.51^a^ | 5.71±0.44^a^ | 76.10±19.61^a^ | 1.59±0.29^a^ | 22.44±3.68^a^ | 30.63±1.68^a^ |
|  | Birch | 0.16±0.03^a^ | 1.44±0.11^a^ | 3.10±0.61^a^ | 9.02±0.77^ab^ | 101.13±21.94^a^ | 3.60±0.40^ab^ | 74.76±21.30^b^ | 111.64±13.92^b^ |
|  | Alder | 1792.11±614.94^b^ | 5.45±1.87^a^ | 10.60±2.94^b^ | 10.09±1.09^b^ | 127.75±29.34^a^ | 5.82±0.95^b^ | 29.28±3.24^a^ | 112.51±14.93^b^ |
| ^1^ Results of two-way ANOVA for the effect of site, species, and its interaction, i.e.: * – significant; - – differences not significant (p < 0.05)  ^2^ Mean ± SE; different letters indicate significant differences within columns | | | | | | | | | |

Table S3. Interaction tree species × site effect on PTEs concentrations in the tree species foliage

| Effect | | Al | Cd | Cr | Cu | Mn | Ni | Pb | Zn |
| --- | --- | --- | --- | --- | --- | --- | --- | --- | --- |
|  |  | [mg kg^-1^] | | | | | | | |
| Species × Site | | - | - | - | - | - | * | - | - |
| PF | Pine | 267.88±16.16^ab^ | 0.46±0.05^b^ | 0.03±0.03^a^ | 5.29±0.30^a^ | 220.04±17.57^a^ | 1.59±0.28^a^ | 0.66±0.07^a^ | 54.16±3.44^a^ |
|  | Birch | 75.83±7.03^a^ | 1.03±0.10^c^ | 0.49±0.14^b^ | 6.48±0.24^b^ | 501.41±21.44^b^ | 1.95±0.22^a^ | 0.96±0.15^a^ | 471.46±25.75^c^ |
|  | Alder | 340.82±116.03^b^ | 0.20±0.03^a^ | 2.11±0.21^c^ | 9.83±0.34^c^ | 231.46±36.29^a^ | 1.58±0.08^a^ | 0.62±0.09^a^ | 114.75±6.02^b^ |
| CP | Pine | 238.37±17.22^ab^ | 0.48±0.07^b^ | 0.01±0.00^a^ | 6.54±0.18^a^ | 360.75±31.65^a^ | 1.76±0.31^a^ | 0.50±0.03^a^ | 54.71±4.79^a^ |
|  | Birch | 86.76±5.21^a^ | 1.07±0.13^c^ | 0.37±0.12^b^ | 9.08±0.55^b^ | 570.96±111.18^a^ | 4.19±0.48^b^ | 1.29±0.24^b^ | 390.35±73.03^b^ |
|  | Alder | 367.23±176.5^b^ | 0.29±0.05^a^ | 1.13±0.17^c^ | 14.76±0.41^c^ | 571.01±100.63^a^ | 3.56±0.41^b^ | 0.78±0.09^ab^ | 126.37±11.29^a^ |
| ^1^ Results of two-way ANOVA for the effect of site, species, and its interaction, i.e.: * – significant; - – differences not significant (p < 0.05)  ^2^ Mean ± SE; different letters indicate significant differences within columns | | | | | | | | | |

Table S4. Interaction tree species × site effect on BAF of studied PTEs concentrations in the fine roots of tree species

| Effect | | BAF of Al | BAF of Cd | BAF of Cr | BAF of Cu | BAF of Mn | BAF of Ni | BAF of Pb | BAF of Zn |
| --- | --- | --- | --- | --- | --- | --- | --- | --- | --- |
|  |  | [mg kg^-1^] | | | | | | | |
| Species × Site | | - | - | - | * | * | - | - | - |
| PF | Pine | 0.00±0.00^a^ | 4.72±1.09^ab^ | 1.41±0.39^a^ | 2.69±0.37^a^ | 3.24±0.50^a^ | 3.32±0.88^a^ | 1.00±0.05^a^ | 4.64±0.40^a^ |
|  | Birch | 0.00±0.00^a^ | 3.11±0.71^a^ | 0.90±0.43^a^ | 3.42±0.48^a^ | 3.66±0.90^a^ | 2.35±1.04^a^ | 1.97±0.38^a^ | 16.57±3.54^b^ |
|  | Alder | 0.28±0.08^b^ | 8.68±1.98^b^ | 1.74±0.56^a^ | 5.65±0.59^b^ | 7.98±1.05^b^ | 1.40±0.41^a^ | 1.04±0.29^a^ | 10.51±2.82^ab^ |
| CP | Pine | 0.00±0.00^a^ | 5.56±0.87^a^ | 0.36±0.09^ab^ | 5.58±1.38^b^ | 9.57±3.34^b^ | 3.27±0.99^b^ | 1.63±0.34^ab^ | 5.74±1.54^a^ |
|  | Birch | 0.00±0.00^a^ | 2.54±0.74^a^ | 0.28±0.08^a^ | 1.80±0.18^a^ | 1.97±0.19^a^ | 0.88±0.24^a^ | 1.92±0.37^b^ | 4.42±1.07^a^ |
|  | Alder | 0.16±0.06^b^ | 6.97±1.65^a^ | 1.15±0.34^b^ | 2.24±0.26^a^ | 2.15±0.28^a^ | 0.95±0.15^a^ | 0.77±0.08^a^ | 2.18±0.37^a^ |
| ^1^ Results of two-way ANOVA for the effect of site, species, and its interaction, i.e.: * – significant; - – differences not significant (p < 0.05)  ^2^ Mean ± SE; different letters indicate significant differences within columns | | | | | | | | | |

Table S5. Interaction tree species × site effect on BAF of studied PTEs concentrations in the tree species foliage

| Effect | | BAF of Al | BAF of Cd | BAF of Cr | BAF of Cu | BAF of Mn | BAF of Ni | BAF of Pb | BAF of Zn |
| --- | --- | --- | --- | --- | --- | --- | --- | --- | --- |
|  |  | [mg kg^-1^] | | | | | | | |
| Species × Site | | * | - | - | * | * | * | - | - |
| PF | Pine | 0.10±0.01^a^ | 1.53±0.34^a^ | 0.01±0.01^a^ | 2.98±0.49^a^ | 16.82±1.31^a^ | 1.29±0.27^a^ | 0.04±0.01^a^ | 6.32±0.69^a^ |
|  | Birch | 0.03±0.00^a^ | 2.36±0.51^a^ | 0.09±0.03^a^ | 3.63±0.61^a^ | 22.76±4.05^a^ | 1.36±0.20^a^ | 0.06±0.01^a^ | 51.86±8.92^b^ |
|  | Alder | 0.16±0.07^a^ | 1.20±0.19^a^ | 3.00±0.79^b^ | 6.42±0.70^b^ | 23.47±3.98^a^ | 2.05±0.19^a^ | 0.05±0.01^a^ | 12.08±2.30^a^ |
| CP | Pine | 0.15±0.05^b^ | 1.86±0.12^b^ | 0.00±0.00^a^ | 6.37±1.51^b^ | 39.84±3.07^b^ | 3.90±1.44^b^ | 0.04±0.01^a^ | 9.80±2.54^ab^ |
|  | Birch | 0.01±0.00^a^ | 1.83±0.48^b^ | 0.03±0.01^a^ | 1.83±0.20^a^ | 13.24±4.04^a^ | 0.99±0.21^ab^ | 0.03±0.01^a^ | 15.42±4.00^b^ |
|  | Alder | 0.03±0.01^a^ | 0.37±0.07^a^ | 0.12±0.02^b^ | 3.29±0.26^a^ | 9.65±0.78^a^ | 0.61±0.11^a^ | 0.02±0.00^a^ | 2.44±0.31^a^ |
| ^1^ Results of two-way ANOVA for the effect of site, species, and its interaction, i.e.: * – significant; - – differences not significant (p < 0.05)  ^2^ Mean ± SE; different letters indicate significant differences within columns | | | | | | | | | |
